# The alteration of interspecific interaction responded to various relative sowing time in wheat/maize intercropping

**DOI:** 10.3389/fpls.2024.1470293

**Published:** 2024-11-05

**Authors:** Jun-Wei Sun, Ying-An Zhu, Yu Pang, Chen-Xi Liu, Jian-Hao Sun, Wei-Ping Zhang, Long Li, Yi-Xiang Liu

**Affiliations:** ^1^ State Key Laboratory for Conservation and Utilization of Bio-Resources in Yunnan, Yunnan Agricultural University, Kunming, China; ^2^ Institute of Soils, Fertilizers and Water-Saving Agriculture, Gansu Academy of Agricultural Sciences, Lanzhou, China; ^3^ Key Laboratory of Plant and Soil Interactions, Chinese Ministry of Education, College of Resources and Environmental Sciences, China Agricultural University, Beijing, China; ^4^ Key Laboratory for Agro-Biodiversity and Pest Control of Ministry of Education, Yunnan Agricultural University, Kunming, China

**Keywords:** sowing time, maximum growth rate, temporal niche, spatial niche, yield advantage

## Abstract

**Introduction:**

An interspecific interaction is an important reason for the yield advantage of interspecific cropping compared with sole cropping, and the relative sowing time of species is an important factor affecting interspecific competitiveness. Our purpose was to explore the effects of different relative sowing times on the interspecific competition-recovery phenomenon in wheat and maize intercropping systems.

**Methods:**

Three planting methods (wheat/maize intercropping, wheat and maize sole cropping) and different relative sowing times of wheat were used to carry out field experiments over two years. Sequential harvest of subplots was performed between 3 and 6 times, and the biomass data were fitted to logistic growth model.

**Results:**

Delaying the sowing time of wheat reduced the wheat yield, biomass and nutrient acquisition and increased those of maize, but wheat still had an intercropping advantage during the co-growth period. At the same time, the nutrient acquisition of maize was still inhibited, but its recovery growth advanced. Changing the relative sowing time of wheat significantly changed the maximum instantaneous growth rates of wheat and maize. Delaying the relative sowing time of wheat significantly reduced its maximum instantaneous growth rate, while enhancing that of maize, leading to a balanced mutual benefit.

**Conclusions:**

Delaying the sowing time of wheat to the same sowing time as maize will change wheat/maize intercropping from asymmetrical interspecific facilitation to symmetrical interspecific facilitation. However, in this case, intercropped wheat still had an interspecific competitive advantage in the co-growth stage, and intercropped maize still underwent a competition-recovery process.

## 1 Introduction

Intercropping refers to the planting method in which two or more crops are planted in different rows or belts during the same growth period. Intercropping is often used in agricultural production to improve the utilisation rate of land and other resources (Asseng et al., 2014). At the same time, the complementary effect generated when two crops are intercropped can further promote the total output (Li et al., 2023). Intercropping can also significantly reduce the occurrence of crop diseases and directly inhibit pathogens through changes in the host physiology (Boudreau, 2013). Patterns of intercropping species are usually developed by farmers over years, which means that not all crop combinations are suitable for intercropping. As a result, the differences between intercropping and sole cropping patterns are often attributed to interspecific interactions between crops.

The promotion and competition of interspecies interactions exist simultaneously in intercropping systems. Interspecific facilitation occurs when the mutual benefit is greater than the competition. In contrast, interspecific competition occurs (Li et al., 2016). Wheat/maize intercropping is a typical model of interspecific competition, and it is widespread in North China because its productivity advantage is achieved through the complementarity of temporal and spatial niches. Previous studies showed that the wheat/maize intercropping pattern limited the growth of maize during the co-growth period (Wang et al., 2022). However, the inhibition of intercropped maize disappeared after wheat maturity and harvest. Subsequently the nutrient acquisition and biomass of maize quickly recovered and reached or exceeded that of sole cropping maize Therefore, wheat and maize had significant intercropping advantages in the field co-growth period (Li et al., 2001a and Li et al., 2001b). The phenomenon of first competition and then recovery in the intercropping system was realised through the interlacing ecological niche interaction after time interlacing, which is known as the “interspecific competition-recovery production principle” (Li et al., 2001a).

The interspecific relationship of species in intercropping is affected by natural conditions and resources such as soil, water, light, temperature, and air, as well as allelopathy and tillage measures (Lithourgidis et al., 2011). For example, neighbouring plants can profoundly affect the biochemical composition of plants in intercropping systems through allelopathy, similar to neighbourhood detection and allelopathy between wheat and 100 other plants through underground signals. Wheat can detect homologous and xenogenic neighbours and respond by increasing allelochemical production, affecting its metabolism and growth (Kong et al., 2018). In intercropping systems, the efficiency of nutrient and water use by crops is often optimised through complementary utilisation. This allows for the effective distribution of soil nutrients and spatial resources among plants, enabling them to coexist more successful (Lithourgidis et al., 2011). For example, the interspecific competition between grains and legumes leads to a disproportionate sharing of soil nitrogen sources. Intercropped legumes fix more nitrogen from the atmosphere than when the legume crop is the sole crop, allowing the intercropping system to utilize more nitrogen resources (Jensen et al., 2020). Therefore, it can be inferred that intercropping can change the utilisation and competition of resources by species through changes in the relative planting time so that the whole intercropping system can gain planting advantages.

The overall advantage of a wheat/maize intercropping system is realised through the complementarity of temporal and spatial niches, so a key factor in the wheat/maize competition-recovery principle is that wheat has an earlier sowing time than maize. This early sowing treatment staggers the period of high nutrient demand for wheat and maize in space and time while meeting the demand for the growing resources for wheat and maize. Therefore, the basis for wheat/maize intercropping to achieve intercropping advantages is that wheat is sown before maize, indicating that the relative sowing time of different crops in the intercropping system is very important for the interaction between crops. For example, maize that was intercropped with cowpea and sown maize in advance promoted the productivity of the whole intercropping system, which disappeared when maize and cowpea were sown at the same time. The sowing time of maize in maize/watermelon intercropping caused different degrees of asymmetric competition and had a great influence on the overall yield (Huang et al., 2018). As a result, the relative sowing time of crop species in intercropping is an important factor affecting the productivity of intercropping systems and it may determine the dominance of different types of interspecies interactions among intercropped crops.

This dynamic trend of interspecific competition within an intercropping system can be analysed using a logistic model. A logistic growth model was established to study the dynamic growth process of plants. The biomass accumulation trend of plants under different growth conditions was compared. For example, fitting the biomass and nitrogen capture data of *Dactylis glomerata* and *Plantago lanceolata* into the logistic model for track prediction showed that there was a strong neighbour effect on transient nitrogen capture in the stem tissues of competing and noncompeting plants (Trinder et al., 2012). In addition, a logistic model was used to quantify and predict the yield and nutrient indexes of intercropped plants, which can be used to make operational adjustments to field agronomic management and to help understand and realize the yield advantages of intercropping (Zhang W. P. et al., 2015).

In conclusion, we speculate that the relative sowing time of wheat is an important factor affecting the “competition-recovery production” phenomenon in wheat/maize intercropping. Earlier sowing of wheat can extend its advantage in intercropping, while maize is able to utilise the extra resources available after wheat harvest to make up the yield, thus influencing interspecific interactions and increasing overall productivity. The objectives of this study are to (1) analyse the effects of the relative sowing time on wheat and maize productivity and interspecific interactions in wheat/maize intercropping, (2) study the effects of different sowing times on crop nutrient acquisition in wheat/maize intercropping, and (3) analyse the growth dynamic curves of wheat and maize intercropping under different sowing times. We hypothesize that earlier sowing of wheat can extend its advantage in intercropping by allowing it to capture nutrients and resources more effectively in the early stages. To fully leverage the advantages of intercropping, it is crucial to understand how agricultural practices influence the growth dynamics and nutrient acquisition of different intercropped species over time. Controlling plant interactions between intercropped crops by adjusting the relative sowing time can promote interspecific facilitation and reduce interspecific competition to improve crop growth and increase the overall yield.

## 2 Materials and methods

### 2.1 Experimental locations

The field experiments were conducted in 2012 and 2013 at the Zhangye Water-saving Agricultural Experiment Station (38°85 ‘N, 100°38’ E) of the Institute of Soil, Fertilizer and Water-saving Agriculture of Gansu Academy of Agricultural Sciences, which is located in the Jiu Gongli Horticultural Experiment Field, 10 km southwest of Zhangye city, Gansu Province, China. The altitude of this place is 1504 m above sea level, the average annual temperature is 7.7°C, the effective cumulative temperatures greater than 0°C and 10°C are 3646 and 3149°C respectively, and the effective cumulative temperature ≥10°C after the wheat harvest is 1350°C. The frost-free period is about 170-180 d, and the number of hours of sunshine is 3023 h. The total annual solar radiation is 5988 MJ m-2 year-1, the average annual rainfall is 150 mm, and the average annual evaporation is 2021 mm, and the adaptive growing period of crops is from the middle of March to the middle of October. The average annual rainfall is 150 mm, the average annual evaporation is 2021 mm, and the adaptive growing period of crops is from mid-March to mid-October, which is a typical continental arid climate ecological zone with a shortage of two seasons and a surplus of one season. All plots were adequately irrigated and hand weeded throughout the crop growth period, and mid-tillage was carried out at appropriate periods of crop growth without any fungicide or insecticide application. Before sowing was conducted in 2013, the topsoil (0-20 cm) contained 16.16 g kg^-1^ organic matter, 0.98 g kg^-1^ total nitrogen, 27.6 mg kg^-1^ available phosphorus, and 93.2 mg kg^-1^ available potassium, and the soil pH value was 7.6.

### 2.2 Experimental design and plant material

**Figure 1:**
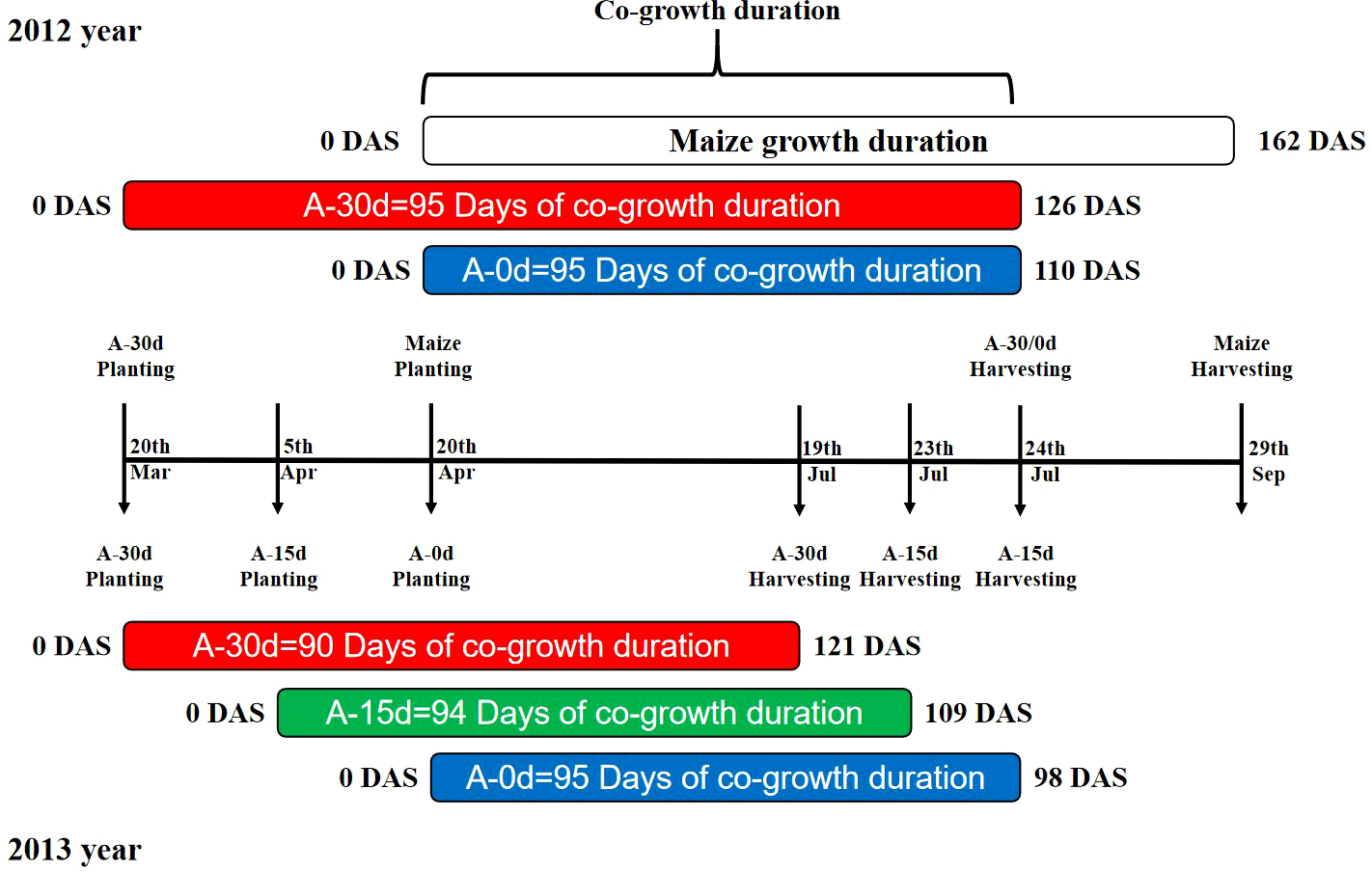
Illustrates the overlapping and co-growing periods in a maize-wheat intercropping system for the year 2012 and 2013. A-30d, A-15d, and A-0d represent different wheat sowing times: A-30d indicates wheat is sown 30 days before maize (normal sowing time for wheat), A-15d indicates wheat is sown 15 days before maize (wheat sowing delayed by half a month), and A-0d indicates wheat is sown simultaneously with maize (wheat sowing postponed by one month). Coloured bars show the duration of wheat growth under different sowing times, while the uncoloured bar represents the maize growth period in the maize-wheat intercropping system.

The experiment was conducted using a two-factor randomised block design with three replicates. The main treatment was the wheat sowing time showed in **Figure 1**. In 2012, two levels were set, namely, 30 days earlier than maize (conventional sowing time and the local soil thawing time) and sowing at the same time as maize (the sowing time was one month later). In 2013, three levels were set: 30 days earlier than maize (conventional sowing time and the local soil thawing time), 15 days earlier than maize (half a month later than normal), and at the same time as maize (one month later than normal). The secondary treatment was the planting mode (sole cropping was abbreviated as S; intercropping was abbreviated as I). The wheat (*Triticum aestivum* L.) cultivar was Longyou No. 2, and the maize (*Zea mays* L.) cultivar was Zhengdan No. 958.

### 2.3 Crop management

**Figure 2:**
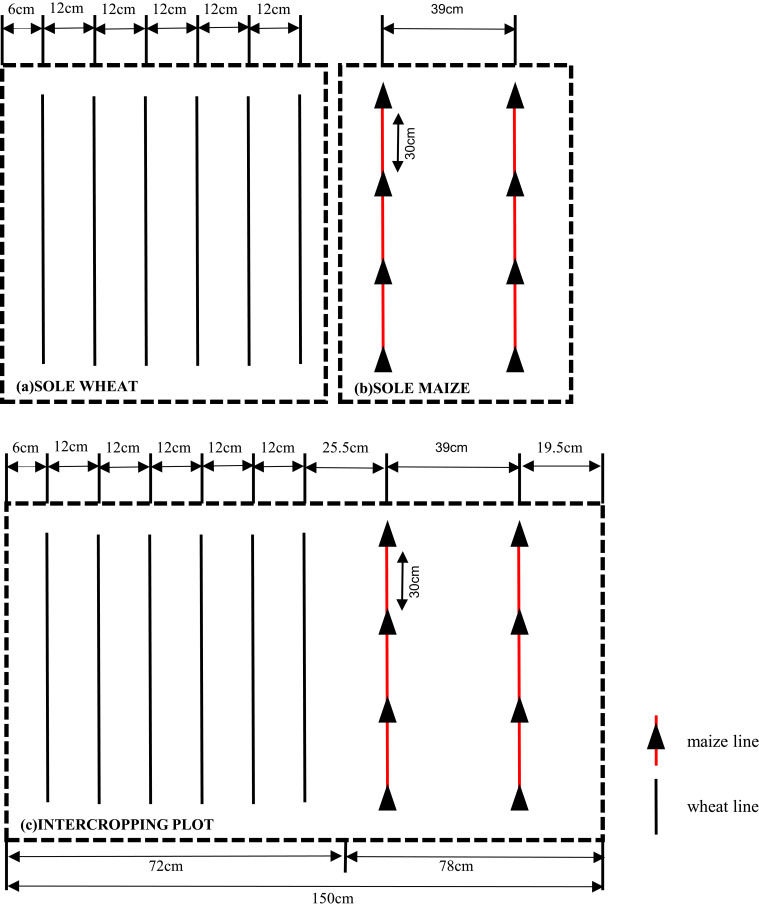
The layout includes sole wheat **(A)**, sole maize **(B)**, and wheat/maize intercropping plots **(C)**. Various lines illustrate the spacing between strips and the distance between individual plants.

In 2012, the plot area was 4.5 m×7.5 m=33.75 m^2^, and three crop combinations were planted. In 2013, the plot area was 6.0×7.5 = 45 m^2^. The row spacing and density of plants planted in the two-year experiments were consistent: each crop combination belt contained 2 rows of maize (row spacing 0.39 m, seed spacing 0.30 m) (**Figure 2B**) and 6 rows of wheat (row spacing 0.12 m) (**Figure 2A**), and the spacing between maize and wheat was 0.255 m (**Figure 2C**). The planting area of maize accounted for 52% of the whole intercropping area, and the planting area of wheat accounted for 48% of the whole intercropping area. The planting density and row spacing of the crops in the sole cropping plots were the same as those in the intercropping plots. The planting density of sole cropping maize was 85,470 plants/ha and that of wheat was 9 million plants/ha. The setting of row spacing and sowing density accords with the actual planting situation of local farmers.

In 2012, 50 kg N ha^-1^ nitrogen fertilizer and 100 kg N ha^-1^ phosphate fertilizer in the form of urea and triple superphosphate, respectively, were incorporated into the surface 20 cm soil as basal fertilizers. In addition, nitrogen topdressing was applied twice during the growth period of maize; that is, 100 kg N ha^-1^ was applied at the jointing stage and the drawing stage of maize, and 100 kg N ha^-1^ was added to wheat at the first topdressing as well as to maize. In 2013, 100 kg N ha^-1^ nitrogen fertilizer and 100 kg N ha^-1^ phosphate fertilizer were turned into the surface 20 cm soil as basal fertilizer in the form of urea and heavy superphosphate. During the growth period of maize, nitrogen topdressing was performed twice in total; that is, 100 kg N ha^-1^ was applied to maize at the joining stage and the pulling stage, while 100 kg N ha^-1^ was added to wheat at the first topdressing. The wheat was given sufficient water and the plot was kept in a healthy state to prevent weed growth. If weeds are found throughout the crop growth cycle, weed control is carried out manually without any herbicides and fungicides.

In 2012, wheat treated 30 days earlier than maize was sown on March 20, wheat treated at the same time as maize was sown on April 20, and both were harvested on July 24; the maize was sown on April 20 and harvested on September 29. In 2013, wheat sown 30 days earlier than maize, sown 15 days earlier and sown simultaneously was sown on March 20, April 5 and April 20 and harvested on July 19, July 23 and July 27, respectively. The maize sowing and harvesting dates were the same as those in 2012 (**Figure 1**). The wheat/maize intercropping was divided into 4 combinations: 1 lateral row area, 2 sampling areas and 1 yield measurement area. The four combined wheat/maize intercropping zones were divided into one side row zone, two sampling zones and one yield measuring zone. The maize was sampled twice after the wheat was harvested. In 2012, samples were collected three times in the co-growth period of wheat/maize: May 27 (wheat flowering stage/maize seedling stage - jointing stage), June 28 (wheat filling stage/maize jointing stage - stag stage), and July 25 (wheat maturation stage/maize despatch stage); the maize was sampled twice after the wheat was harvested: August 27 (maize filling stage) and September 29 (maize maturity stage). In 2013, samples were collected four times in the co-growth period of wheat/maize: May 18 (wheat heading stage/maize seedling stage), June 18 (wheat flowering stage/maize jointing time), June 30 (wheat filling stage/maize large trumpet stage), and July 14 (wheat ripening stage/maize tapping stage), and the maize was sampled twice after the wheat was harvested: August 19 (maize filling stage) and September 20 (maize maturity stage). In addition, to better fit the logistic model curve, the sampling time points of wheat biomass increases were as follows: May 5, June 11, 2012; June 2 and July 30, 2013.

### 2.4 Sample collection

Sampling method: When sampling the intercropped wheat, we started from the side row of the sampling belt and collected wheat in an area of 30 × 30 cm from the outside to the inside (that is, 3 rows with a length of 30 cm). For sole-cropped wheat, the same area as that used for intercropping was selected for sampling. In addition to 8 maize plants selected in the first sampling, 4 maize plants were collected in the sampling area according to the progressive method in other growth periods to determine the dry matter and nutrient concentrations of the plants. At crop maturity, both the biological yield and grain yield were measured. From the measurement zone, ten maize plants and twenty wheat plants were selected for seed testing, then dried and crushed to analyse plant nutrients. The zone was harvested to determine biomass and grain yield, with the biomass data used to fit a logistic growth function.

### 2.5 Statistical analyses and growth model

The land equivalent ratio (LER) is often used to measure the intercropping advantage of an intercropping system, and LER is defined as the area of sole cropping needed to achieve the same yield as intercropping (Mead 1980). The calculation formula is as follows:


(1) $$\text{LER}=\frac{\text{YintercroppedA}}{\text{YsolecroppedA}}+\frac{\text{YintercroppedB}}{\text{YsolecroppedB}}$$

Y_inter croppedA_ and Y_inter croppedB_ represent the yields of crops A and B in intercropping according to the area occupied, and Y_solecroppedA_ and Y_solecroppedB_ represent the respective grain yields of crops A and B in sole cropping. When LER > 1, the same land area of intercropping can produce a higher yield than that of single cropping, which means that the intercropping system has higher resource utilisation efficiency.

Logistic models can characterize the growth of plants from emergence to harvest, so they are increasingly used to adapt to the dynamic trend of plant growth. Biomass data for each component of all harvested sole and intercropped crops were matched by a logistic growth function using the least square method (2)


(2) $$M\text{t}=\frac{K}{1+\text{exp(}r\times (t50-t\text{))}}$$

where *M*_t_ (kg ha^−1^) is the ground dry matter weight per unit area of a particular crop grown under different treatments t days after sowing. *K* (kg ha^−1^) is the parameter representing the maximum biomass, *r* (d^−1^) is the initial growth rate, and *t*_5_ (d) is the time for the maximum instantaneous growth rate. These parameters were calculated using the Slogistic1 procedure of Origin 2023 software (OriginLab Corporation, Northampton, MA, USA).

The instantaneous growth rate can be estimated as:


(3) $$\frac{dMt}{dt}=rMt(1-\frac{Mt}{K})$$

The instantaneous growth rate reaches its maximum value at Mt=K/2, so the maximum instantaneous growth rate Imax=rK/4 occurs at t50.

The effects of different relative sowing times and sole/intercropping patterns on the biomass and nutrient acquisition data of wheat and maize were analysed. The yields, LER, nutrient uptake and RLD in different sowing time and planting pattern were analysed by one-way or two-way analysis of variance (ANOVA). Analyses were carried out in the PASW Statistics 18 software (SPSS Inc., Chicago, IL, U.S.A.) and mean values (n = 3) were compared by least significant difference (LSD) at the 5% level. Origin 2023 software was used for mapping and logistic model curve fitting.

## 3 Results

### 3.1 Effect of the relative sowing time on plant grain yield

The grain yield of intercropped wheat was significantly higher than that of sole cropping wheat regardless of the sowing time. The biological yield of intercropped wheat significantly decreased with the delay of the sowing time in 2013 but showed no difference in 2012 (**Table 1**). However, intercropped wheat still had an advantage over sole cropping in terms of yield, increasing by 22.7% and 25.5% in 2012 and 2013, respectively, when wheat and maize were sown simultaneously. The grain yield of maize was greatly affected by the delay in wheat sowing. When maize was intercropped with wheat, the wheat sowing time was delayed by 30 days, increasing grain yield by 37.7% in 2012 and by 13.9% in 2013. Under the traditional sowing time treatment, there was no significant difference in grain yield between maize intercropped with wheat sown 30 days in advance and sole-cropped maize. However, when wheat sowing was delayed by 30 days, the grain yield of intercropped maize increased by 28.3% and 23.8% in 2012 and 2013, respectively, compared to that of sole cropping maize. In addition, the LER of wheat/maize intercropping was not consistent in the two years and significantly increased with the delay of wheat sowing in 2012 but showed the opposite trend in 2013.

**Table 1:** Grain yield and LER of wheat and maize in sole cropping and intercropping systems under different sowing times.

Days before maize sowing		2012			2013			
		30 d	0 d	Mean	30 d	15 d	0 d	Mean
Grain yield (kg ha ^-1^)	IW	3556a	3383a	3469A	6679a	4989b	4409b	5070A
	SW	2796a	2858a	2827B	3965a	3654a	3604a	4041B
	IM	16333b	22485a	19409A	21271b	22701b	24222a	22731A
	SM	17526		17526B	19564			19564B
LER	—	1.09b	1.24a	1.16	1.37a	1.25ab	1.23b	1.32

“I” stands for intercropping, “S” for sole cropping, “W” for wheat, and “M” for maize. Different days of treatment refers to the number of days before the maize was sown. Values are means of 3 replicates. Values followed by the same lowercase letters are not significantly different among different sowing times of wheat within the same cropping system for the same species at the 5% level based on the LSD (horizontal comparison); values followed by the same capital letters are not significantly different between different cropping systems for the same species at the 5% level based on the LSD (vertical comparison).

### 3.2 Effect of sowing time on the biomass accumulation dynamics of wheat/maize sole cropping and intercropping

**Figure 3:**
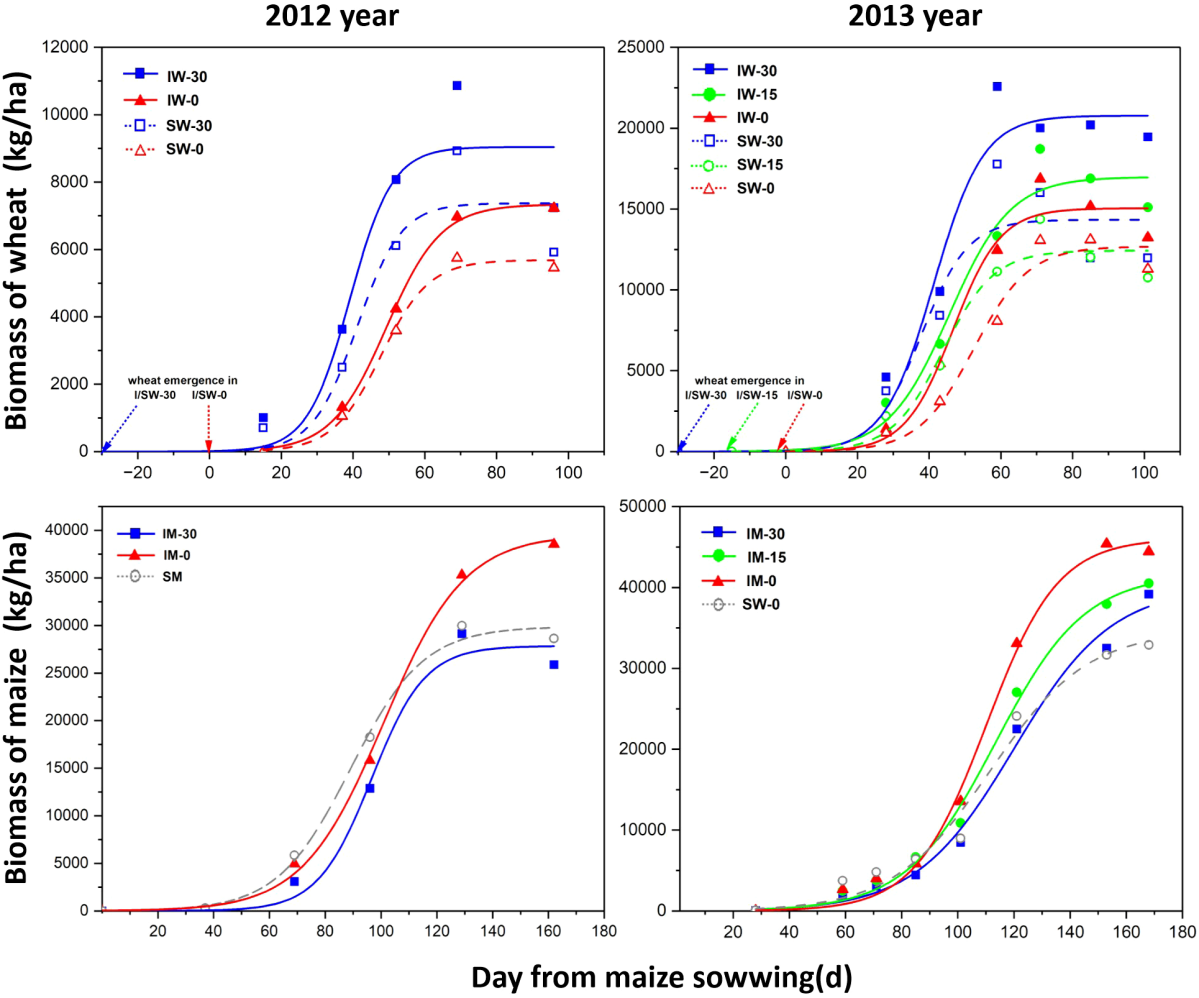
Effects of different relative sowing times and planting patterns on crop biomass. Aboveground biomass growth of wheat and maize in relation to cropping treatments and wheat sowing time. Each symbol represents a single harvest and is the mean of three replicates. “I” stands for intercropping, “S” for sole cropping, “W” for wheat, and “M” for maize. “−30”, “−15”, and “−0” indicate the number of days in advance relative to the maize sowing time.

The biomasses of wheat and maize under different treatments were consistent with the logistic model (**Figure 3**; **Table 2**). The biomass accumulation of intercropped wheat was higher than that of sole-cropped wheat, reaching its maximum value approximately 60 days after the maize sowing. At the same time, the initial growth rate and maximum biomass of intercropped wheat were higher than those of sole cropping wheat in both 2012 and 2013.

**Table 2:** Parameters estimated from fitting logistic growth curves to wheat and maize biomass growth in relation to the wheat sowing time.

Year	Treatments		Biomass				
			*r*	*K*	*t_50_*	*I_max_*	Adj. R-Square
			(×10^3^ d^-1^)	(t ha^-1^)	(d)	(kg ha^-1^ d^-1^)	
2012	Wheat	IW-30	0.169 ± 0.151	9042.90 ± 1186.05	39.12 ± 4.63	381.93	0.86284
		IW-0	0.131 ± 0.011	7339.82 ± 145.21	49.26 ± 0.72	239.74	0.99757
		SW-30	0.162 ± 0.121	7372.46 ± 1003.23	41.08 ± 5.05	299.08	0.85986
		SW-0	0.148 ± 0.028	5684.96 ± 238.60	47.79 ± 1.49	210.67	0.98753
	Maize	IM-30	0.104 ± 0.046	27854.51 ± 1663.89	96.62 ± 3.12	724.91	0.97738
		IM-0	0.069 ± 0.006	39576.18 ± 1005.35	100.90 ± 1.64	682.39	0.99749
		SM	0.076 ± 0.011	29883.64 ± 997.51	89.05 ± 2.19	570.12	0.99285
2013	Wheat	IW-30	0.145 ± 0.060	20775.84 ± 1321.84	41.31 ± 2.95	752.09	0.93716
		IW-15	0.117 ± 0.041	16980.42 ± 1221.66	45.66 ± 3.46	495.28	0.94338
		IW-0	0.150 ± 0.056	15047.38 ± 958.81	46.65 ± 2.88	564.65	0.95318
		SW-30	0.153 ± 0.108	14339.07 ± 1590.19	37.75 ± 5.44	548.36	0.79586
		SW-15	0.140 ± 0.065	12432.57 ± 947.16	43.82 ± 3.50	434.21	0.92448
		SW-0	0.130 ± 0.038	12688.10 ± 854.74	52.42 ± 2.94	411.98	0.9718
	Maize	IM-30	0.057 ± 0.009	40044.80 ± 2735.67	119.93 ± 4.38	570.12	0.98702
		IM-15	0.063 ± 0.007	41675.76 ± 1591.25	113.45 ± 2.45	654.73	0.99269
		IM-0	0.078 ± 0.009	46048.50 ± 1364.21	110.19 ± 1.76	903.01	0.99327
		SM	0.058 ± 0.012	34648.97 ± 2550.89	111.51 ± 4.91	498.69	0.97384

“I” means intercropping, and “S” means sole cropping. “W” stands for wheat and “M” for maize. “-0”, “-15”, and “-30” represent the number of days before the sowing time of wheat relative to that of maize. r(d-1) is the initial growth rate, K (t ha-1) is the asymptotic maximum biomass, t50(d) is the time to reach the maximum instantaneous rate, and Imax (kg ha-1 d-1) is the maximum instantaneous growth rate. The mean ± standard error calculated from three biological replicates are shown.

Intercropping and the timing of wheat sowing significantly impacted the initial growth rate of maize (**Table 2**). The initial growth rate (*r*) of wheat decreased as the sowing date was delayed. In contrast, the *r* value for maize decreased in 2012 but increased in 2013. Additionally, the time required for wheat to reach its maximum instantaneous growth rate (*t50*) extended with later sowing, while maize *t50* shortened in 2012 and lengthened in 2013 as wheat sowing was delayed.

The maximum growth rate (Imax) of intercropped wheat was greater than that of sole-cropped wheat. The Imax of wheat decreased with a later sowing time, and although the Imax of wheat sown 15 days later in 2013 was lower than that of wheat sown 30 days later, the overall *I_max_* trend decreased. The *I_max_* of maize decreased in 2012 with the delay of wheat sowing but showed an upwards trend in 2013.

**Figure 4:**
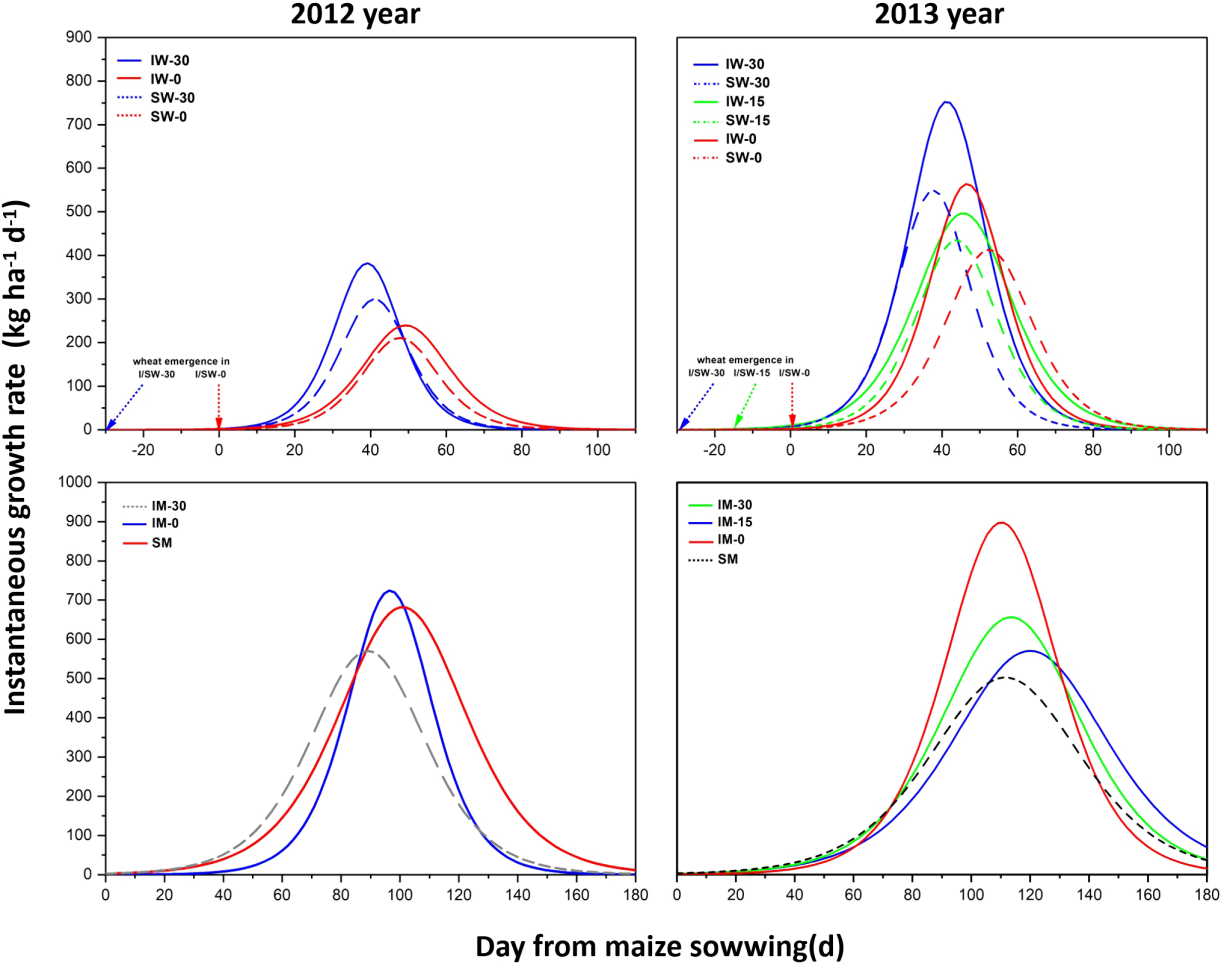
Instantaneous growth rates of wheat and maize in relation to cropping treatments and the relative sowing time of wheat. “I” means intercropping, and “S” means sole cropping. “W” stands for wheat and “M” for maize. “−0”, “−15”, and “−30” represent the number of days before the sowing time of wheat relative to that of maize.

The logistic model predicted that the instantaneous growth rate of wheat intercropped with maize was higher than that of sole-cropped wheat, and the maximum instantaneous growth rate of intercropped wheat was higher due to early sowing. However, the maximum instantaneous growth rate of wheat sown at the same time (IW-0) was lower than that sown 30 days in advance (IW-30) and higher than that sown 15 days in advance (IW-15). Wheat that was sown early reached the instantaneous maximum growth rate faster than wheat was sown at the same time as maize (**Figure 4**), but sole cropping or intercropping had no significant effect on the time to reach the instantaneous maximum growth rate.

In the 2-year field experiment, the variation tendency of the instantaneous growth rate of maize was not consistent (**Figure 4**). In 2012, the maximum instantaneous growth rate of maize intercropped with wheat sown 30 days in advance (IM-30) was higher than that of maize intercropped with wheat sown simultaneously (IM-0), but the difference was not significant. In 2013, the maximum instantaneous growth rate of maize intercropped with simultaneously seeded wheat (IM-0) was the highest, which was significantly higher than that of the other treatments. The maximum instantaneous growth rate of intercropped maize decreased with the delay in wheat sowing. The data from two years of experiments proved that the maximum instantaneous growth rate of intercropped maize (IM) was higher than that of sole cropping maize (SW).

### 3.3 Acquisition of nitrogen, phosphorus and potassium

**Figure 5:**
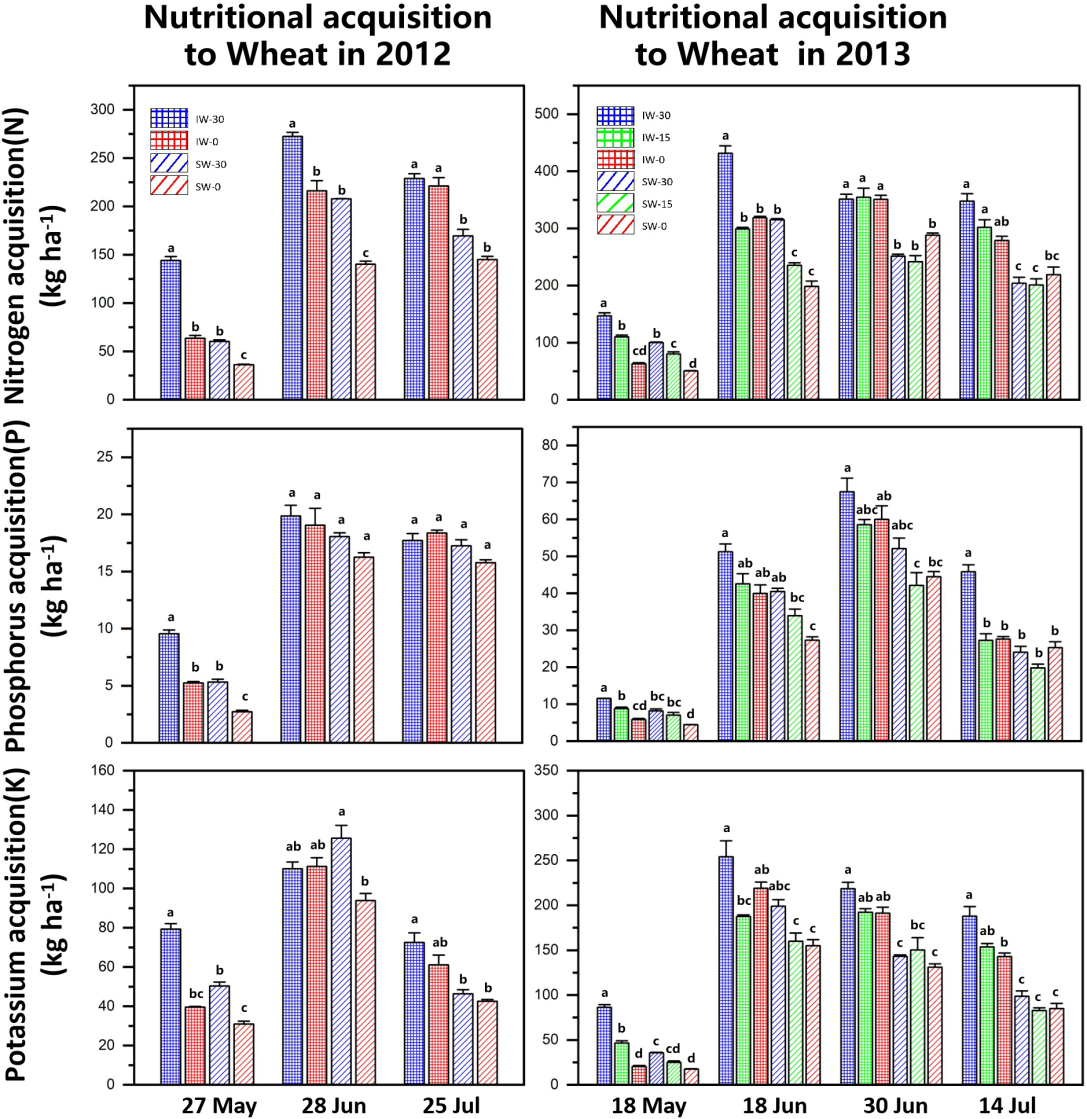
Nutrient acquisition of intercropped and sole-cropped wheat under different sowing times. “IW” indicates intercropped wheat, “SW” indicates sole-cropped wheat, and “−30”, “−15” and “−0” indicate the number of days that wheat was sown ahead of maize. Values are means of 3 replicates. Bars of standard error with different lowercase letters indicate significant differences among nutrient acquisitions at the same growth stage (P<0.05).

The nitrogen absorption of wheat first increased and then decreased and reached its highest value in June. The nitrogen acquisition of intercropped wheat (IW) in all treatments was higher than that of sole-cropped wheat (SW), and the nitrogen acquisition decreased with the delay in the sowing time. This occurred in both years of the field experiments. At the same time, wheat that was sown early (IW-30 and SW-30) had significantly higher nitrogen acquisition in the early co-growth stage, but there was no significant difference in the filling stage (**Figure 5**).

The changes in the phosphorus acquisition of wheat were basically the same as those of nitrogen acquisition, which also showed a trend of increasing first and then decreasing and reached the highest in June. In the 2012 experiment, the phosphorus acquisition of wheat decreased with the delay in the sowing time, but there was a significant difference only in May. In 2013, the phosphorus acquisition of wheat decreased significantly with the delay in the sowing time. The phosphorus acquisition of intercropped wheat was higher than that of sole-cropped wheat.

The overall potassium acquisition of intercropped wheat was significantly higher than that under sole cropping, but in 2012, sole-cropped wheat that was sown 30 days in advance (SW-30) reached its maximum value in June, which was greater than that of intercropped wheat (IW). After the peak in June, wheat potassium absorption decreased significantly and decreased with the delay in the sowing time.

**Figure 6:**
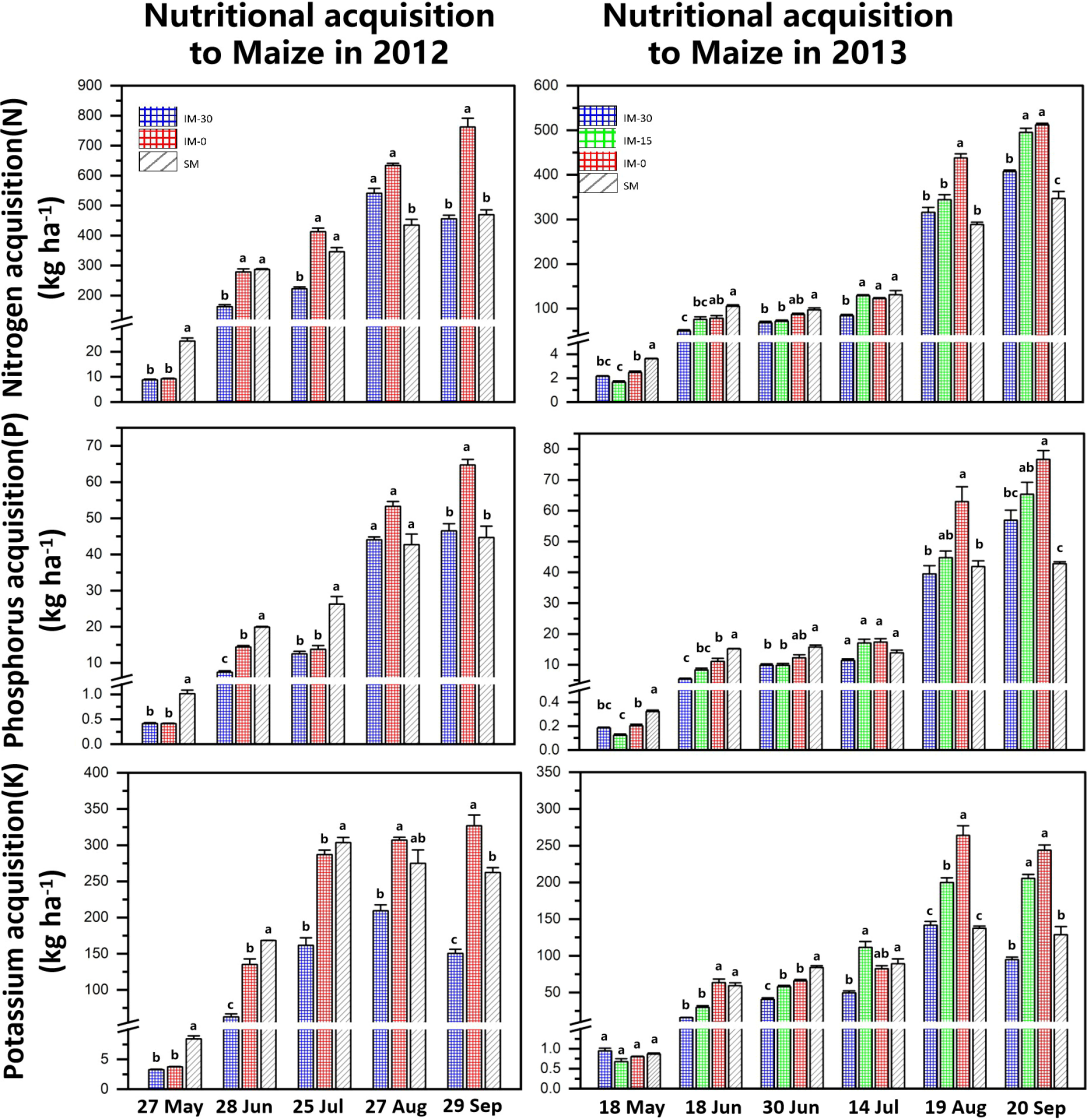
Nutrient acquisition of intercropped and sole cropping maize at different sowing times. “IM” indicates intercropped maize, “SM” indicates sole-cropped maize, and “−30”, “−15” and “−0” indicate the number of days that wheat was sown ahead of maize. Values are the means of 3 replicates. Bars of standard error with different lowercase letters indicate significant differences among nutrient acquisitions at the same growth stage (P<0.05).

The nitrogen acquisition of maize increased significantly with the shortening of the relative sowing time and peaked before the harvest in September. Under wheat/maize intercropping, the nitrogen acquisition of maize increased with the delay in the wheat sowing time. In the 2012 experiment, the nitrogen acquisition of maize sown at the same time as wheat (IM-0) was higher than that of wheat sown in advance (IM-30), and the difference was significant. The 2013 experiment confirmed this trend. At the same time, the nitrogen acquisition of intercropped maize (IM) was lower than that of sole-cropping maize (SM) in the early stage but higher than that of sole-cropping maize in the late stage (**Figure 6**).

The change trend of phosphorus acquisition in maize was consistent with that of nitrogen acquisition and reached the maximum value at harvest. In the early co-growth period of wheat/maize (May to July), the phosphorus acquisition of sole cropping maize (SM) was higher than that of intercropped maize (IM). However, with the maturation of wheat, the phosphorus acquisition of intercropped maize was reversed at the end of the intercropping co-growth period. The phosphorus acquisition of IM was significantly higher than that of SM in August–September after the wheat harvest. At the maize harvest, the phosphorus acquisition of maize sown at the same time as wheat (IM-0) was significantly higher than that of other treatments, while the phosphorus acquisition of intercropped maize with wheat sown 30 days in advance exceeded (IM-30 in 2013) or approached (IM-30 in 2012) the phosphorus acquisition level of sole cropping maize (**Figure 6**).

The changes in the potassium acquisition of maize were basically the same as those in the nitrogen and phosphorus acquisition of maize and showed a trend of increasing gradually with the growth period of maize. At the same time, the potassium acquisition of intercropped maize (IM) was lower than that of sole cropping maize (SM) in the intercropping co-growth stage and higher than that of sole cropping maize (IM) in the later growth stage after the wheat harvest. Maize’s potassium uptake increased significantly as the relative sowing time of wheat was delayed. At the maize harvest, the potassium acquisition of IM-0 in both years was significantly higher than that of SM. The potassium acquisition of intercropped maize in 2013 was higher than that of sole cropping maize overall, but the potassium acquisition of intercropped maize (IM-30) in 2012 was lower than that of sole cropping maize (**Figure 6**).

## 4 Discussion

### 4.1 The relative sowing time transforms the interspecific interaction of intercropping

A difference of 15 or 30 days in sowing time can significantly alter the interspecific interaction. The sowing time of crops is a key step in agricultural production that significantly affects crop adaptability and grain yield (Abbas et al., 2019; Wang et al., 2021). In the present research, intercropping significantly increased the grain yield of wheat while maintaining that of maize at the traditional sowing time (**Table 1**), indicating that wheat/maize is an intercropping model based on wheat advantage, in accordance with Li’s definition of an asymmetric interspecific interaction (Li et al, 2001a). This type of interspecific interaction also appears in wheat/peas (Vályi-Nagy et al., 2024), maize/soybean (Zhang Y. T. et al., 2015), and maize/faba bean (Zhang et al., 2024) intercropping. According to local farming practices, intercropped wheat is sown one month before maize, which makes a significant contribution to the greater competition of wheat than maize (Liu et al., 2015 and Liu et al., 2020). The yield advantage of intercropped wheat was significantly decreased with the delay of the wheat sowing time, but both intercropped wheat and maize had greater grain yield than that under sole cropping with the same sowing time (**Table 1**). These results indicated that delaying the sowing time of wheat relative to maize could indeed improve the interspecific competitive ability of maize relative to wheat and translate asymmetric interspecific interactions into interspecific facilitation. This may be due to the delayed sowing time of wheat reducing its competition for root-zone nutrients and moisture with maize during the growing period, as well as decreasing the shading effects of wheat on maize. Consequently, the reduced competition between wheat and maize allows the complementary ecological niches of the two crops, such as root distribution, nutrient uptake, and ventilation, to become more evident. In the okra/maize and okra/cowpea intercropping models, delaying the sowing time of okra also significantly reduced the yield of okra (Muoneke et al., 1997). This effect on yield occurs because differences in planting dates can alter the competitive balance between intercropping species (Andersen et al., 2007).

In addition, the LER of wheat/maize intercropping under the traditional sowing time showed obvious differences between 2012 and 2013. These results may be due to the significantly higher rainfall in 2013 compared to 2012, which increased the yield advantage of intercropped wheat. This result also indicates that the practice of sowing wheat 30 days earlier than maize can demonstrate a more significant productivity advantage in environments with more abundant resources.

### 4.2 The adjustment of the competition-recovery mechanism with various relative sowing times

Traditional wheat/maize intercropping exhibits symbiotic competition and maize recovery after wheat harvest (Li et al., 2001a and Li et al, 2001b). The results of this study in 2012 and 2013 showed that the biomass and nutrient acquisition of intercropped wheat were significantly higher than those under sole cropping when sown 30 days in advance during the whole growth period, while those of intercropped maize were significantly lower than those under sole cropping during the co-growth period and quickly recovered to the same level as those under sole cropping after the wheat harvest (**Figures 2**–**4**). The results showed that wheat/maize intercropping under the traditional sowing time showed an obvious competition-recovery phenomenon, which is consistent with the results from previous studies (Liu et al., 2015; Zhang W. P. et al., 2015). Delaying the sowing of wheat significantly reduced its biomass and nutrient acquisition in the intercropping system, highlighting that early sowing is a key factor contributing to wheat’s advantage in intercropping (Gou et al., 2016). However, even if wheat and maize were sown at the same time, the biomass of intercropped wheat was still higher than that under sole cropping. These results indicate that wheat/maize intercropping has other competitive advantages in addition to spatial and temporal niche complementarity.

In addition, interestingly, delaying the wheat sowing time to be consistent with that of maize improved the competitiveness of maize and consequently, wheat/maize intercropping appeared to be mutually beneficial. However, nutrient uptake in intercropped maize was lower than in monocropped maize during the early stages of co-growth. As growth progressed, nutrient uptake of intercropped maize recovered to the same level as that of monocropped maize and even exceeded that of monocropped maize after wheat harvest. These findings suggest that even when wheat and maize are sown at the same time, the wheat-maize intercropping system still exhibits a ‘competition-recovery’ dynamic.

### 4.3 Relative sowing time significantly affected the growth dynamics of the intercropped plants

The growth dynamic curves of the crops provide a clear insight into the impact of interspecific interactions on crop growth (Trinder et al., 2012; Wei et al., 2020). In this study, the intercropping pattern and the relative sowing time of wheat showed great effect on the maximum biomass, the maximum growth rate and the time for the maximum instantaneous growth rate of wheat and maize. Adjusting these factors effectively altered crop growth dynamics, underscoring the importance of optimizing sowing timing and intercropping arrangement. Our results revealed that delaying the sowing time of wheat significantly reduces the maximum growth rate of wheat, while increasing the maximum growth rate of corn (**Table 2**; **Figure 4**). These results effectively highlight the changes in competitive abilities between wheat and maize under different sowing times in intercropping systems. The variation in maximum growth rates may also be a significant factor contributing to the shifts in interspecific interactions within the wheat/maize intercropping system. In faba bean/wheat intercropping, the intercropping system exhibited a significantly higher initial growth rate and maximum instantaneous growth rate in both faba bean and wheat, contributing to greater yield and biomass accumulation (Luo et al., 2021; Li et al., 2014). Additionally, compared to monocropping, intercropped maize and wheat exhibited a higher maximum instantaneous growth rate across all sowing times, which is consistent with their biomass and nutrient accumulation patterns.

The growth rate, which reflects a plant’s ability to grow and the resource availability, is a key indicator of a plant’s ability to compete with other plants (Tilman, 2020). In the current study, wheat exhibited a higher initial growth rate than maize across all sowing times and planting patterns. Additionally, wheat reached its maximum instantaneous growth rate within 35-50 days, whereas maize reached its peak between 90–120 days (**Table 2**; **Figure 4**). These results suggest that wheat has a significantly stronger growth and developmental capacity during the early stages of the intercropping period, which is the fundamental reason for its greater competitive ability compared to maize. Therefore, the fact that plants occupy the soil first is an important factor affecting interspecific competition. In general, earlier-sown plants gain advantages in accessing light and space resources. As the sowing time of intercropped plants is delayed, resource competition will vary according to environmental conditions. Therefore, in intercropping systems, it is essential to take into account both the characteristics of intercropped species and the environmental factors when determining sowing time, to reduce interspecific competition and enhance mutual facilitation (Ma et al., 2020; Wu et al., 2022).

## 5 Conclusions

Delaying the relative sowing time of intercropped wheat can change the interspecific relationship in wheat/maize intercropping, and asymmetric interspecific facilitation can be transformed into symmetrical interspecific facilitation. Despite the delays, the intercropping system continued to exhibit a ‘competition-recovery’ dynamic, even as maize recovered earlier than before. Optimising sowing time based on the maximum instantaneous growth rate of the crop is essential for managing interspecific competition. Thus, by rationalising sowing time, interspecific competition can be reduced and early recovery can be accelerated, resulting in a significant increase in crop yield and economic returns. This optimisation approach can be used as a reliable method to improve the efficiency and sustainability of different intercropping methods.

## Data Availability

The original contributions presented in the study are included in the article/supplementary material. Further inquiries can be directed to the corresponding authors.
